# Report of a Case of Appendicitis without Preceding Pain or Fever1Read at the twenty-eighth annual meeting of the Central New York Medical Association, held at Syracuse, October 15, 1895.

**Published:** 1896-01

**Authors:** S. L. Elsner

**Affiliations:** Rochester, N. Y.


### REPORT OF A CASE OF APPENDICITIS WITHOUT PRECEDING PAIN OR FEVER.[1]

By S. L. ELSNER, M. D., Rochester, N. Y.

MR. A. H., *æt.* 32, German, clothing cutter. On July 6th, in the forenoon, I found him complaining of nausea with a distress in the epigastric region. He said that during the night he awakened and felt that his night lunch had been too much for him. He vomited twice during the early morning hours and was positive that if he could be made to vomit again he would feel relieved. Examination of his abdomen was negative, his temperature was normal and pulse 74. I prescribed pepsin and soda and did not expect to see him again. The same afternoon, however, he sent for me. I found him in just the same condition excepting that he appeared more impatient and nervous. I

1. Read at the twenty-eighth annual meeting of the Central New York Medical Association, held at Syracuse, October 15, 1895.

gave him one-eighth grain of morphia, hypodermically, after which he rested two hours. At night he again summoned me and I found him with no pain, but still with the single symptom of distress in the stomach. His bowels had moved, temperature 98°, pulse 78. Upon making firm pressure over the cecum he had slight tenderness. There was, however, no soreness or pain after the examination. July 7th, at 9 A. M., I found my patient having a severe chill, his temperature now was $100\frac{4}{5}$° and pulse 96. He presented an anxious expression, but denied having the slightest pain ; he stated that the distressed feeling which he so bitterly complained of on the evening preceding had entirely left him and he had a good night's rest. Examination at this time elicited much more tenderness on pressure over McBurney's point, and in view of his chill and tenderness I diagnosticated appendicitis and advised immediate exploration. He consented and I returned for him in about one hour. He walked out, placed himself in my wagon and I drove him to the hospital. At no time during the ride, and I frequently asked him, did he complain of pain. He was prepared in the usual manner and I operated that same evening. The appendix was found much enlarged, gangrenous and ready to rupture at its base. There were very few adhesions and the operation was rapidly completed. The patient made a good recovery without having had a particle of pain and never but the single rise of temperature of $100\frac{4}{5}$ during the chill.

I report this case because I consider it rather unique and of great practical value in diagnosticating some of these rapid and often fatal cases of appendicitis. The fact of there being no adhesions might possibly account for the absence of pain. It also demonstrated how rapidly fatal such cases would be were they not early recognised and quickly operated upon.

Given a patient with local tenderness over the cecum and an anxious expression, regardless of temperature, pulse and local pain, appendicitis should be suspected. A chill, with the above, followed by a rise of temperature and pulse, call for immediate exploration. I place more reliance upon a chill as indicating beginning perforation than any one single symptom and have learned to waste no time in getting into the abdomen in such cases.

The specimen which I will pass about for your inspection is about one-half its original size, but illustrates very lucidly how much destruction can take place in a few hours.

DISCUSSION OF DR. ELSNER'S PAPER.

Dr. W. S. CHEESMAN, Auburn : For the sake of introducing the discussion, I would like to say that, in some respects, the case seems to me quite unique. In this case the patient complained not at all of pain at any stage of the illness. I do not think it is rare to see cases of appendicitis that have gone on to perforation in which there is no pain. That is one of the commonest things, I think, and one of the most deceptive. You cannot depend at all upon pain, the most reliable sign being the condition of pulse and general appearance of the patient. Here is a case of appendicitis going on to gangrene, and yet the patient has no pain whatever, except a slight one in the epigastrium. I think it asserts the importance of early operation. I myself find I am coming more and more to the view that if you can make the diagnosis of appendicitis you are justified in operating. Suppose you make an error: in that case you have not performed a dangerous operation. You have made an opening of the abdomen, to be sure, but if it is not appendicitis you close the opening after having made an aseptic incision. If it is appendicitis you save the life of the patient. No surgeon or physician, I think, can tell us we are face to face with this trouble. We are not absolutely sure and do not know whether that case will go on to perforation; we do not know whether it will limit itself to catarrhal inflammation or subsequently advance. But we must decide promptly and either perform the operation or say in so many words, "I don't know" and wait and see what happens.

Dr. N. S. Jacobson, Syracuse: I feel, with Dr. Cheesman, this is a subject of too much importance to pass by without remark. During the past four or five years those of us who have been called to meet these cases surgically have had perhaps a varied experience. I can say I have frequently encountered cases where there has been absence of characteristic symptoms upon which we can depend. I have found the pulse and temperature in some cases normal. I have met several times a case without pain but with localised tenderness, usually at the characteristic point, but to find a case of perforated appendicitis without pain or tenderness is a condition which I have not encountered. I have always been able to make out a characteristic point of fixed localised tenderness.

In a case seen during the month of July with Dr. Sears, the pains started in the back, and the diagnosis was that of lumbago. At my first visit, on the eighth day of the sickness, there was an area of tenderness so extensive that it was no longer difficult to recognise the fact that a most serious condition of things existed. Upon operation we found a large post-cecal abscess. There existed also, in this case, the unusual complication of a secondary entirely separate appendicular abscess, in which the gangrenous appendix lay separated from the intestine.

I have found that it is not wise to depend upon the temperature or the pulse. Usually we find rigidity of the muscles, however, and evidences of trouble can be made out by a careful study of local symptoms

The author of the paper lays great stress upon the occurrence of the chill as indicative of perforation. Upon this point I should disagree with him, recognising rather the chill as significant of a septic condition than as warning us of the occurrence of a perforation. Inasmuch as most of the cases of appendicitis are now considered as due to infection by the bacillus coli communis, we are forced to realise that we are dealing with a septic condition. I have felt that I could depend in my diagnosis of the occurrence of perforation rather upon the manifestations of shock.

As to the question of operation, we are all agreed that the earlier it can be performed the better are the results obtained. While I am by no means ready to maintain that every case of appendicitis demands surgical intervention, I am satisfied that these cases which show progression after the first twenty-four hours are more safely treated by surgical than medical means. In, however, that class of cases in which the septic manifestations are so rapidly progressive that general peritonitis is almost a coincidence with the appendicitis, no surgical operation apparently is of avail.

A case seen with Dr. Elsner, of Syracuse, presented the history of a man who had gone to his work, as usual, at seven o'clock in the morning and at ten o'clock called at the office of the doctor, at which time it was already possible to diagnosticate general septic peritonitis. Although there has recently been made in our medical journals a claim for the cure of septic peritonitis by means of irrigation of the abdominal cavity with sterilised salt solution, I must confess that in my hands this line of treatment has not as yet been satisfactory.

As to the case presented by the writer of the paper, I feel that he is to be congratulated upon his recognition of the existing condition, for as a result of his diagnostic acumen the case terminated happily, while, had the operation been delayed for another twenty-four hours, I believe there would have been found associated with appendicitis general septic peritonitis, with a probable fatal ending.

Dr. C. O. Baker, Auburn : I want to congratulate the author upon his diagnosis. It is a question in my mind if there can be a case of appendicitis entirely without pain. My experience with these cases leads me to believe that there is always accompanying pain in some portion of the abdomen. The inflammation may be very moderate, it may be severe ; circumstances control it. That condition may be passing over forty-eight hours, possibly longer than that, without pain or any indication that there is any difficulty about the patient except the nausea that is caused by the probable absorption going on.

To open up the whole subject of appendicitis requires time. The symptoms of it are indefinite; so, too, are the landmarks for operation, as well as the time, the exact time an operation shall be done. I hold that no operation should be done for appendicitis until we are satisfied that we have an appendicitis. I hold, further, that there is no such thing as a harmless opening of the walls of the abdomen. I do not care how skilful the operator, he cannot repair those parts as perfectly as they were before. Now, the patient has a right, in appendicitis, of traveling round without the possibility of a hernia, without the possible following of any weakening of the walls of the abdomen ; that right is a grave right which any physician should consider and should consider with weight.

Dr. A. B. MILLER, Syracuse : One thought that impresses me in listening to the discussion, one that specially regards the diagnosis, and one to which I would take exception, is that in any question of doubt exploratory incision should be resorted to, under our present antiseptic methods, and no serious consequences would attend the procedure, while possibly a valuable life would thereby be saved. There is a belief in the profession at present that pain does attend such cases and that pain may be relieved under anesthetics. In our practice, however, it need not be necessary to resort to the exploratory method. After the patient is thoroughly anesthetised, the abdominal walls relax, and palpation alone should clear up any question of doubt as to its being normal or otherwise. In my office practice I palpate the appendix in all my cases, differentiating the normal from the abnormal ; thus, by palpation, the existence of appendicitis can be apprehended before opening the abdomen.

Dr. S. L. ELSNER, of Rochester: The gentlemen who have so kindly discussed my paper have simply reiterated what I stated in it. I would state, for the benefit of some who perhaps misunderstood me, that I did not say that this man had no tenderness. He did have tenderness, but at my second visit it was so slight I did not feel that I was going to encounter any trouble. Upon the second visit this tenderness was more marked, and aside from that tenderness there was absolutely no physical sign. Of course, with that tenderness, followed rigidity of the muscles over the right side of the abdomen, which we could not get from any other condition. There was no other sign present to lead me to suspect appendicitis. The fact he had a chill, the fact the temperature went up, his pulse went up, the expression of the man's face—my past experience has taught me to look at such patients as seriously ill—with such symptoms I have found it always has been a wise thing to explore. The distress that he spoke of was in his epigastrium ; it was not pain. The man expressed himself in German. Upon being asked in German if it was a bad pain, he said, "No, it is not a pain, simply an uneasiness in my chest; if I had something to empty my stomach I would feel all right."

When I said perforation, I meant an inflammation had taken place.

When perforation takes place it is caused by the processes of softening, due to the inflammation and the ulceration, which leads to the perforation. In connection with the diagnosis made in this case, I would like to bring to your notice another case in which I made just as positive a diagnosis as I did here, and the man had every symptom of appendicitis. He had a chill. I opened that man's abdomen ; I found the appendix septic for three inches, caused by gangrene. I am positive the man never would have recovered if I hadn't opened him. I know sometimes these patients after operation are often not as well as they were before, as far as strength of the abdominal wall is concerned, but they are certainly given a chance they wouldn't have had if operation had not been resorted to. In concluding I would say that cold water injections are not efficient in my hands.

[The discussion on the papers of Drs. Hall and Snow was not received in time for the December issue, hence is printed here.]

DISCUSSION OF DR. HALL'S PAPER.

Dr. N. S. Jacobson, Syracuse : Unfortunately I was not present at the beginning of the paper ; hence, in discussing this very interesting subject, cannot speak as to the statements of the writer. However, I am convinced that these cases are extremely rare, and many of the books state it is impossible to make an absolute diagnosis of this condition during life. Most of the cases have been discovered after death and, generally speaking, fracture of the greater tuberosity is usually associated with dislocation of the head of the humerus.

As to the treatment, it has been very unsatisfactory until recently, when, I believe, foreign surgeons have suggested that this is particularly a place for the open treatment. Prior to the antiseptic era it was, of course, considered a very serious procedure to treat a compound fracture openly, as fully fifty per cent. of the cases died ; but we have reached a point in antiseptic procedure when we may, as a matter of choice, open down upon the fractured surface, and in this case particularly the line of treatment that is to be pursued in the future is the open treatment. I think it promises as well here as any line of treatment, and I feel satisfied that the writer has obtained as good a result in his case as possible, except perhaps by the open treatment.

Dr. A. L. Hall, Fair Haven : I wish to reply to one statement made by Dr. Jacobson—namely, that nearly all were diagnosticated after death. No doubt that is true in many instances, but they have been diagnosticated beyond a question when alive and three or four other observers beside myself have recorded cases of undoubted fracture of the greater tuberosity of the humerus. The open line of treatment in old persons I think would not be wise, unless there was displacement.

Dr. T. H. Halsted, Syracuse: One thing which occurs quite frequently in early tubercular involvement of the larynx is anemia of the soft palate. A marked anemia of the uvula and soft palate is a symptom which though, of course, it occurs in other diseases, should at once suggest beginning tubercular disease of the lower respiratory tract, and this symptom is none the less valuable because it is an early one.

Morell Mackenzie makes the statement, in his book published in 1884, that up to that time he had seen but four cases of tubercular laryngitis cured. The literature of today is full of cases of healed and cured tubercular ulcerations and infiltrations of the larynx. This improved prognosis is due to the improved local treatment of the disease—the treatment of Krause and Heryng—the local application of lactic acid in varying strengths and, in selected cases, the curettement of the larynx, followed by the rubbing in of lactic acid. Lactic acid is the best local application we have, whether we hope to effect cicatrisation of the ulcer or merely give our patients temporary relief from their dysphagia.

Then, with reference to the Adirondacks, we should exercise great care and good judgment in selecting cases to be sent away from home. Some we send away hoping the disease may be arrested and the patient cured; others, again, for whom the best to be hoped for is a prolongation of life. Advanced cases, and especially patients who are unusually nervous, are only likely to advance more rapidly because of the deprivations of home comforts, lack of food suitable for a sick person with a capricious appetite, and the noise and confusion incident to a hotel or boarding-house. Ordinarily, we can look for improvement in patients in the first and second stages, and these persons, with of course exceptions, can be sent there. We should not be satisfied to simply say, "Go to the North Woods." We ought to be able to tell them just where to go and, if possible, to know of some good boarding-houses. Many, especially men, do much better to keep clear of the regular boarding houses and hotels, and live literally in the woods. Many patients whom I have sent to the Adirondacks have improved most wonderfully, the disease being arrested in some only to recur later, and in others there has been no recurrence. I have seen extensive tubercular ulcerations in the larynx heal and cicatrise under the application of lactic acid and a residence of some months in the Adirondacks. I have great faith in the North Woods, but discriminate in sending patients, and, believing in the local treatment of tubercular laryngitis, make it a point to send patients requiring local treatment to some part of the woods where such treatment can be had. The Saranac region, the region of Lake Placid and North Elba meet these requirements, and I endeavor to send my patients there, though believing no one section has any very material advantage over any other, except for the reasons named. I also believe that patients do better there in the winter than in the summer months.

Dr. J. P. Creveling, Auburn: In some of these cases where the ulcerative process has closed and the mucous membrane reformed, if you examine carefully you will find the tissues underneath and around remain infiltrated and thick, so that in reality the disease is not entirely removed. The mucous membrane remains in a tuberculous condition, so to speak, and the disease is liable to start anew. There is no doubt but that this condition may remain dormant for years and, possibly, the disease be regarded as removed, when in reality it is only held in check. A number of such cases have come under my observation during the past five or ten years. I now recall a case I treated some fifteen or sixteen years ago that I supposed had recovered, but she presented herself to me again last summer after having traveled all over the United States and having been treated here and there at intervals, as occasion required, only for me to discover the throat lesions reappearing and a dulness at the apex left of the lung. The disease had been held at bay all this time. It is sometimes a question just how much permanent benefit is received by sending some of these cases away for only a time, for on their return home the disease again brightens up and its progress is equally as rapid as before.

One word regarding the anemic condition referred to about the palate and throat, as in my opinion that depends very much upon the general condition of the individual, especially in the case of girls or young women. I have had those cases come to me in years past, where the throat presented this pale, anemic appearance, with a slightly troublesome cough without any tangible evidence of tubercle anywhere and I have seen those same girls grow up, in fact, they appear on our streets today, developed into strong, vigorous women. I do not regard the anemic condition as characteristic—merely suggestive.

Dr. H. L. Elsner, Syracuse: The discussion, as it stands, leaves the subject under consideration in a hazy and rather unsettled condition, which fact prompts me to say a few words. I must take exception to the statements made by Dr. Creveling, for I do not believe that the disease returns with renewed energy after the patients with tuberculosis return from the Adirondacks. The disease may not be arrested in some of these cases, the disorganising processes may not have been influenced materially, or the cases may not have been fit ones for treatment in the woods. There are cases of tuberculosis which cannot be benefited by a climatic change. Many such have been sent to the Adirondack region. These must necessarily return to have the disease make the usual progression towards the fatal ending. We cannot expect permanent relief in the majority of cases, but it is certain that the experience of many careful clinical observers justifies the conclusion that there are cases of pulmonary tuberculosis, as well as tuberculous laryngitis, which do well in the Adirondacks and which continue to do well after their return. At best, climatic treatment must be largely experimental, for there are no rules which we can follow in directing our patients. It is within the experience of all physicians to find cases with similar symptoms and the same pathologic changes differently affected by the same climate. Our deductions must be reached after a thorough consideration of the results in a large number of cases. The class of cases which is most benefited by a stay in the Adirondacks is not of the acute type of the disease. Such cases we recognise by the high temperature, rapid pulse and progressive emaciation. The physical signs are not always well marked, for the tuberculous process is disseminated and disorganisation takes place only after the disease has existed for a few weeks or months. On the other hand, with infiltrating tuberculosis, where the disease is not far-reaching, the temperature not above 102° at night and the pulse below 100, you will find the benefits of the Adirondacks positive in the majority of cases. It is necessary for these patients to be kept under observation, and I know of no place where this can be better done than in the North Woods. I recall at this time a number of cases, which have been practically cured by a prolonged stay and the treatment instituted at the sanitarium at Saranac. I do not mean to say that the tuberculous has been changed to normal lung tissue, but I am positive that the disease has become latent. The patients are now enjoying comparative health and have become useful members of society. Tuberculosis can become latent, and it is not an infrequent experience to find a considerable area of latent tuberculosis in adults who are to all appearances perfectly healthy.

I have seen so many good results follow timely visits to the Adirondacks in well-selected cases and at the proper season, that I have been forced to look upon this region as one of the greatest boons which the people of this state enjoy. Unfortunately these woods, with their hundreds of lakes and lofty health-giving pines, have not been appreciated by the people, otherwise we would not be pained to see the destruction of large tracts for the purposes of gain and the various other evidences of vandalism, by which we will soon be robbed of a tract which ought to be preserved and at once converted into a national or state park. It is the duty of the profession to impress these facts upon the laity, as well as upon our legislators, that steps may be taken to insure protection.

Dr. J. P. Creveling, Auburn: If the doctor will remember, I did not say the ulcers reopened, yet I believe they may, or rather that new ones may take their place. I said the mucous membrane might remain infiltrated and new ulcers appear at any time. Now, we have all seen some of these cases get well at home. I have made and Dr. Elsner undoubtedly more often, dissections where there has been found evidence of former lung lesions such as old cicatricial tissue, the result, probably, of healing of an abscess or some other structural change. Such cases live among us today. I am not speaking against the Adirondacks or of a particular class of cases—in Dr. Snow's paper he made distinction—it was a general assertion of tuberculosis regardless of class I am still convinced of the truth of my former statement and that this is true of any place, the Adirondacks being no exception.

Dr. W. J. HERRIMAN, Rochester: I would like to say that in conversation with Dr. Trudeau, last spring, at Saranac Lake, he said to me that a majority of cases of incipient tuberculosis should not be sent to the Adirondack region during the early spring or summer, and Dr. Elsner brought that remark to my mind when he said that in well-selected cases he was a firm believer in the efficacy of climatic treatment. I would add that the patient should be sent there at some definite time of the year. Dr. Trudeau, whom we look to as authority, gave me the impression that cases could be sent there in the fall or early winter in preference to any other time of the year; that cases sent there during the spring and summer did not, as a rule, do well until the advent of cold weather, but cases sent there during the fall and winter did well then and continued to improve during the subsequent summer. The particular case in question that was talked of, a lady, was at that time in Asheville, N. C., and Dr. Trudeau advised her to remain there until the fall, until the close of warm weather, and then have her take up her residence in the Adirondack region, depending more upon the rarity and dryness of the atmosphere than upon the altitude alone.

Dr. F. S. CREGO, Buffalo: If Dr. Cheesman will take the chair a moment, I would like to say a word concerning the treatment of tubercular troubles. As regards location, I have no very important suggestions to offer on the subject, but I have had a very unpleasant experience in sending friends, or advising them to go to the Adirondacks. I do not know whether any other gentlemen have had the same experience. I had two cases last year that I sent there. One died, a very bright physician; and another physician, who did not improve very much, went to Denver. It seems to me that the only treatment of these cases is to get them into a high altitude and keep them there. In a very limited experience, that has been my observation. There are a few cases that recover in the Adirondacks, but a great many more recover in higher altitudes, in the West and in Asheville, N. C. I would like to hear from some gentlemen here as to their experience as regards climate. It seems as though a great many of these cases were wasting time in going to the Adirondacks; it is not a high enough altitude, not dry enough, it seems to me, in a vast number of instances. Of course Dr. Snow has had experience. My limited experience has been just the opposite from Dr. Snow's.

Dr. S. F. SNOW: There is nothing in particular that I would add, except that I want to make myself plain, and the paper I think would have done so if I had been able to finish it. I do not advise sending advanced cases to the Adirondacks. I think the cases in the first and in the beginning of the second stage of tuberculosis should be sent there. In regard to high altitude, I do not attach the importance to it that Dr. Crego does. I think that more depends upon the soil; we must be sure of good drainage, a dry pure air; altitude in many cases has not seemed to avail very much. I think patients should be up from 1,000 to 1,200 feet.

Another point that I think we ought to emphasise is that the patients should take their systematic bathing, cold baths I mean, and exercise out of doors, exercise every day they can get it.

